# Characterization of afferent corpuscular sensors of the human palmaris brevis muscle

**DOI:** 10.1111/joa.13098

**Published:** 2019-11-07

**Authors:** Silvia Bramke, Christian Albrecht May

**Affiliations:** ^1^ Department of Anatomy Medical Faculty Carl Gustav Carus TU Dresden Dresden Germany

**Keywords:** distribution, immunohistochemistry, palmaris brevis muscle, striated muscle

## Abstract

The palmaris brevis muscle contains numerous muscle spindles to control changes of the muscle length but is devoid of tendon‐associated neuronal elements (e.g. Golgi tendon organs or Ruffini‐like corpuscles) controlling changes in muscle strength. Pacinian bodies, frequently seen in the palm of the hand, show no direct association to the muscle bundles. The observed innervation pattern of the palmaris brevis muscle points to a specific type of neuronal regulation, present in skeletal muscles with no skeletal connection.

## Graphical abstract

The palmaris brevis muscle reveals a distinct number of fiber bundles, each containing numerous muscle spindles. It shows no tendon‐associated neuronal elements but some surrounding Pacinian bodies.

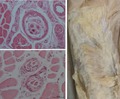

## Introduction

The human palmaris brevis muscle is a constant superficial muscle without a separate muscle fascia running transversely to the hypothenar eminence. Two recent publications in this journal described the fiber type composition of the palmaris brevis muscle (primarily type I muscle fibers; Moore et al. 2017) and discussed functional implications defining the muscle as an active protector of the neurovascular structures within the ulnar canal (Moore & Rice, 2017). Another recent publication added some macroscopic data on the palmaris brevis muscle appearance and topography (Kim et al. 2017).

Since striated muscles show a complex innervation pattern which is crucial for their functional integration, the present work focused on the hitherto unknown presence and distribution of corpuscular sensors in the human palmaris brevis muscle to estimate the muscle's responsiveness to mechanical stimuli.

## Materials and methods

### Tissue preparation

Muscle specimens of the palmaris brevis muscle were collected from 19 human caucasian cadavers. They were part of the donor program of the Department of Anatomy in Dresden (Germany) and had given in their lifetime written consent for use of their bodies for the purpose of science and education after death. Nine male and 10 female cadavers, age range between 77 and 105 years, lacked general neuro‐ or myopathies in their medical history as far as documented. All cadavers were fixed 2–4 days postmortem with a mixture of formalin and alcohol and remained in that solution for at least 1 year. After dissecting the epidermis and dermis and identifying the margins of the palmaris brevis, the muscle was removed with its surrounding connective tissue and washed several times in phosphate‐buffered saline (PBS, pH 7.4, 0.01 m).

### Histology and immunohistochemistry

From tissue embedded in paraffin wax, serial sections (5 μm thick) of each specimen were cut and selected sections stained with hematoxylin and eosin (H&E) to identify muscle spindles and the general morphology of the muscle. Additional sections were stained with a Sirius red solution to better distinguish between cytoplasmatic (light green) and extracellular (red to dark green) tissue.

For immunohistochemistry, muscle specimens were washed in PBS and longitudinal 20‐μm‐thick frozen serial sections were cut. The sections were frozen for 3 days, then air‐dried for 3 h at room temperature. The sections were incubated with normal horse serum for 30 min at 37 °C. The primary antibody against human neurofilament (antibodies‐online ABIN378774, diluted 1 : 100) was added and incubated overnight at 4 °C. After washing in PBS, an appropriate biotinylated secondary antibody was added and incubated for 15 min at 37 °C, followed by washing and incubation with a VECTASTAIN® Elite ABC mouse kit (PK 6102 Vector Laboratories Inc., Burlingame, CA, USA). Visualization of peroxidase activity was realized by adding 3,3‐diaminobenzidine for 8 min.

The sections were examined on a Zeiss Jenamed2 microscope (Carl Zeiss AG, Oberkochen, Germany) and images were recorded using a Digital Sight DS‐Fi1 camera (Nikon AG, Tokyo, Japan).

The complete right palmaris brevis muscle of four donors was sectioned and the number and type (simple/compound/tandem according to Watanabe & Suzuki, 1999) of muscle spindles counted.

## Results

**Figure 1:**
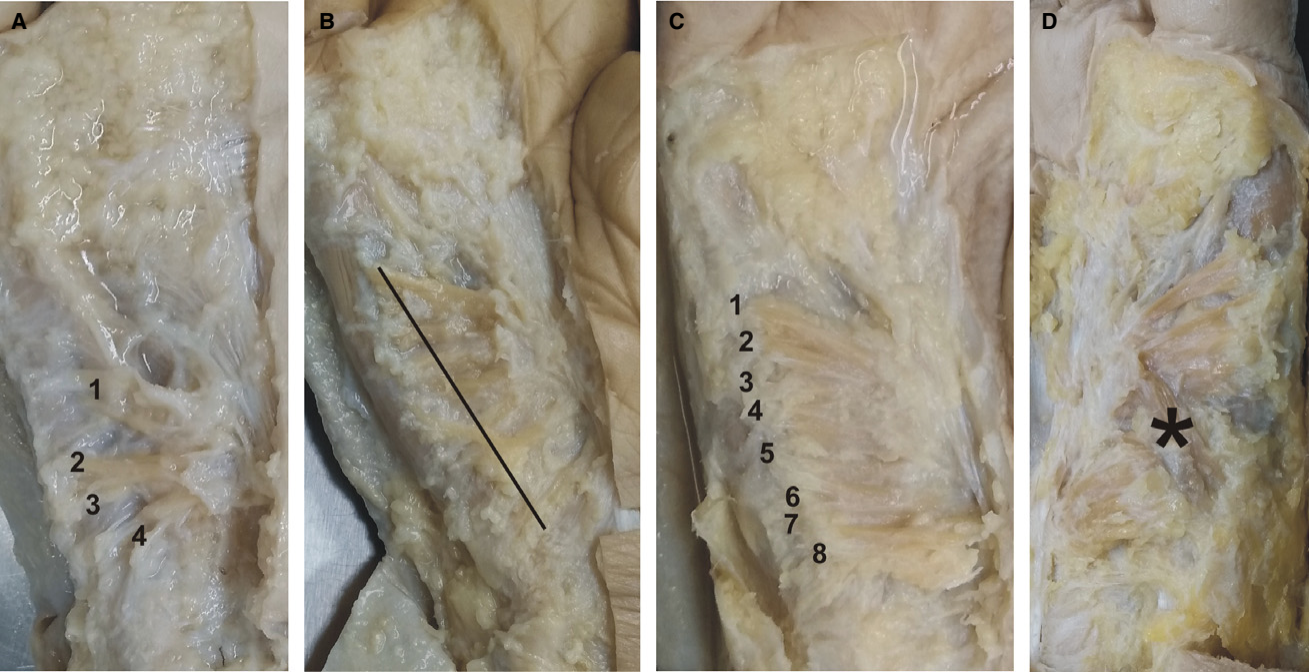
Examples of different palmaris brevis muscle appearances (micrographs show the location between the pisoscaphoid line at the bottom and the palmar digital crease at the top). (A) An 89‐year‐old female with a small right palmaris brevis muscle revealing four fiber bundles. The muscle is restricted to the proximal part. (B) An 82‐year‐old female with a fan‐shaped right palmaris brevis muscle (line marks the width of 30 mm). (C) A 92‐year‐old male with eight parallel‐oriented palmaris brevis muscle fiber bundles (right hand). (D) An 88‐year‐old male with an especially wide left palmaris brevis muscle revealing some unusually oriented fiber bundles in the middle of the muscle (asterisk).

The palmaris brevis muscle could be easily identified in the subcutaneous layer. In most cases (13 of 19), the mean mid‐width of the muscle was 34 mm (SD ± 4 mm; range 29–40 mm), the mean length of the muscle fibers was 24 mm (SD ± 3 mm; range 20–28 mm); single data are listed in Table 1. One male donor (88 years of age) showed an especially wide palmaris brevis muscle (48 mm on the left, 55 mm on the right side) well beyond the normal range (25 mm on the left, 28 mm on the right side; Fig. 1D). Four female donors showed a smaller muscle with a mean of 20 mm in width (SD ± 4 mm; range 15–26 mm) and an almost normal mean length of 22 mm (SD ± 4 mm; range 18–28 mm; Fig. 1A). One male donor (77 years of age) showed also a shorter muscle width (20 mm on the left, 18 mm on the right side; length: 27 mm on the left, 28 mm on the right side) but at the distal end of the muscle some atrophic fiber bundles were visible in both hands, pointing to a degenerative process in this case.

**Table 1:** Data of the donors and palmaris brevis width (from proximal to distal) and length (from radial to ulnar) of the hands investigated

Age and sex	Right hand			Left hand		
	Width (mm)	Length (mm)	No. of fiber bundles	Width (mm)	Length (mm)	No. of fiber bundles
89 years f	19	19	4	19	19	3
95 years f	23	25	4	24	25	4
105 years f	17	28	4	15	21	4
89 years f	15	18	6	26	20	8
77 years m	*18*	*28*	*4*	*20*	*27*	*4*
82 years f	**30**	**20**	**9**	**30**	**22**	**8**
90 years f	**34**	**22**	**7**	**31**	**22**	**6**
94 years f	**35**	**22**	**7**	**34**	**20**	**7**
93 years f	**37**	**26**	**7**			
95 years f	**30**	**23**	**7**			
98 years f	**35**	**28**	**9**			
84 years m	**32**	**20**	**8**	**38**	**21**	**9**
77 years m	**29**	**30**	**6**	**28**	**27**	**6**
83 years m	**40**	**28**	**7**	**40**	**27**	**7**
92 years m	**34**	**26**	**8**	**33**	**24**	**7**
88 years m	**30**	**28**	**9**	**35**	**24**	**7**
69 years m	**35**	**28**	**8**			
78 years m	**32**	**24**	**8**			
88 years m	55	28	7	48	25	7

The macroscopic distinguishable muscle bundles at the ulnar side are listed as no. of fiber bundles. The bold numbers refer to the ‘normal’ reference situation. The cursive numbers showed pathological signs of atrophy.

f, female; m, male.

The macroscopic organization of the palmaris brevis muscle revealed a distinct number of fiber bundles at the ulnar origin condensing somewhat towards the palmar aponeurosis. The mean number of fiber bundles was 7.5 ± 1 for the majority group (Fig. 1C; Table 1); the small‐muscle group showed a mean of 4.6 ± 1.6 fiber bundles (Fig. 1A; Table 1).

The shape of the muscle showed some interindividual variations, ranging from completely parallel (Fig. 1C) to a fan‐shaped (Fig. 1B) arrangement of the fiber bundles. Only rarely were ‘chaotic’ orientations of fiber bundles noted (Fig. 1D). There were no major differences between the left and the right hands of a single donor.

At both ends of the muscle, the fiber bundles merged into tendon‐like collagen bundles connecting the fibers with the hypothenar muscle fascia on the ulnar side and with the palmar aponeurosis on the radial side. The individual muscle fibers were not directly connected to the dermis but via subcutaneous retinacula.

### Muscle spindles in the palmaris brevis muscle

**Figure 2:**
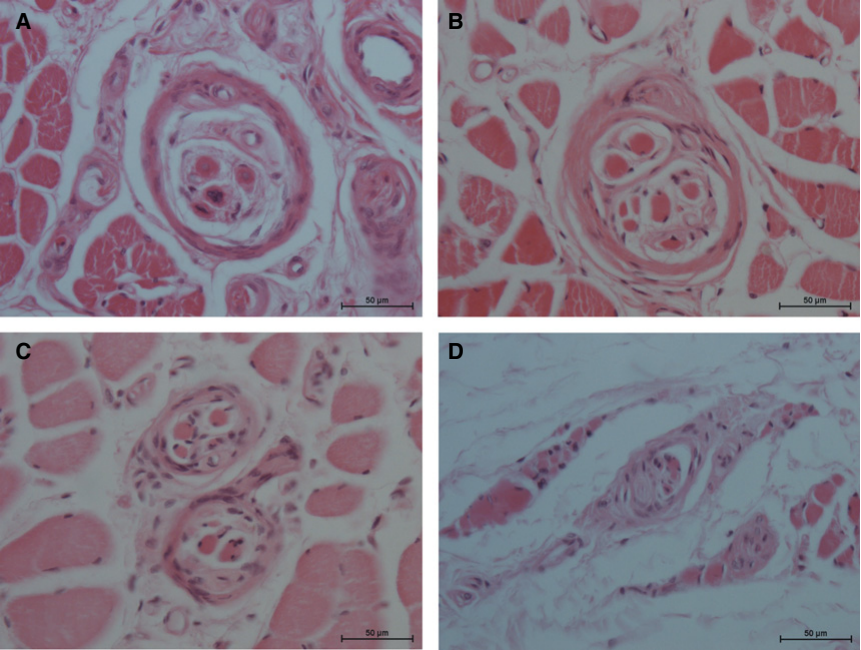
Muscle spindle appearance in the palmaris brevis muscle (HE‐staining). (A) Simple type muscle spindle with characteristic morphology (98‐year‐old female donor). (B) Compound type muscle spindle with only a thin separation between the individual intrafusal fiber groups (77‐year‐old male donor with normally developed palmaris brevis muscle). (C) Compound type muscle spindle with complete individual sheaths (82‐year‐old female donor). (D) Atrophic muscle spindle (77‐year‐old male donor with partial palmaris brevis muscle atrophy).

Serial sections through the palmaris brevis muscle revealed that all macroscopically distinguishable fiber bundles contained two to six muscle spindles. The total number of muscle spindles in three palmaris brevis muscles of different donors with normally developed palmaris brevis muscles were 18, 19, and 26 spindles. In the 77‐year‐old male donor with atrophic fiber bundles, the total number of muscle spindles was 13. Interestingly, the atrophic parts still contained single atrophic spindles (Fig. 2D).

The muscle spindles were mainly located in the middle of the muscle bundles’ length; the associated spindles ended as soon as the tendon‐like connective tissue appeared at the rim of the muscle bundles. The topographic location of individual spindles within the cross‐sections varied: most spindles were located within the muscle bundles, and some spindles were located in the loose connective tissue between muscle bundles.

Concerning the muscle spindle types, roughly two‐thirds of all muscle spindles belonged to the single type (mean: 67.6 ± 5%; Fig. 2A), and a substantial number were compound muscle spindles (mean 32.4 ± 5%; Fig. 2B,C). Some of the compound muscle spindles had a y‐shaped appearance with a common region on one side, whereas others ran parallel to each other along the whole length. Tandem type muscle spindles were not observed in our samples of the palmaris brevis muscle.

Using the published weight of 0.9 g for a normally developed palmaris brevis muscle (Voss, 1971), the predicted number of muscle spindles according to Banks (2006) was 12.4 muscle spindles and the mean relative abundance of the actual measured number of muscle spindles in the palmaris brevis muscle was 1.7.

The light microscopic cross‐sectional appearance of the muscle spindles showed the main characteristics of a two‐layered capsule and a neuronal and vascular supply (Fig. 2A). Although the donors were all of higher age, the spindles showed a normal appearance and only mild thickening of the capsule was noted.

**Figure 3:**
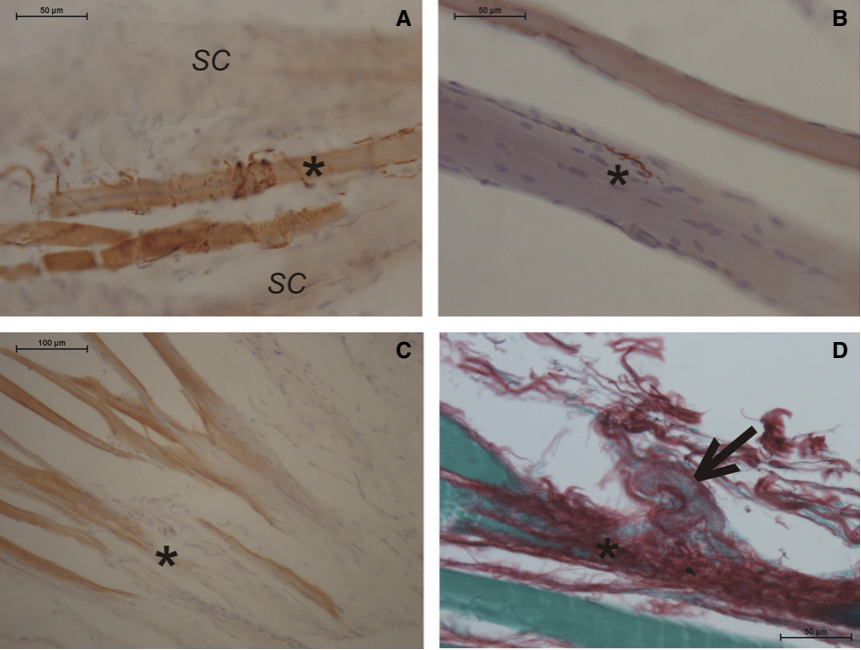
(A) Example of the annulo‐spiral innervation of a nuclear chain fiber (asterisk) of a muscle spindle; SC marks the capsule of the spindle. (B) Motor end plate (asterisk) of an extrafusal muscle fiber. (C) At the musculo‐tendinous junction (asterisk) no stained nerve fibers are visible in the whole region. The muscle fibers reveal a non‐specific background staining. (D) A suspected nerve aggregation (arrow) next to the musculo‐tendinous junction (asterisk) which could not be confirmed by immunohistochemistry: (A–C) immunohistochemical staining for neurofilament; 90‐year‐old female donor; (D) Sirius red staining; 78‐year‐old male donor.

Longitudinal sections of the muscle spindles stained with the general neuronal marker neurofilament showed the annulo‐spiral innervation of the intrafusal muscle fibers (Fig. 3A). Some nerve fibers were also seen at the extrafusal muscle fibers forming motor end plates (Fig. 3B).

Differentiating the intrafusal muscle fibers, most muscle spindles showed one or two nuclear bag fibers and between two and nine nuclear chain fibers.

### Tendon‐associated organs in the palmaris brevis muscle

Serial sections through the tendon‐like regions of the palmaris brevis muscle did not show any specific neuronal aggregations pointing to the presence of Golgi tendon organs. In addition, no nerve fibers or nerve endings were detected within the tendon‐like connective tissue or at the musculo‐tendinous junction (Fig. 3C). Even some suspicious cellular or fibrillary aggregations occasionally seen in the Sirius red staining (Fig. 3D) could never be confirmed to contain neuronal elements, indicating sensory corpuscular structures (e.g. Ruffini‐like corpuscles).

### Pacinian bodies near the palmaris brevis muscle

**Figure 4:**
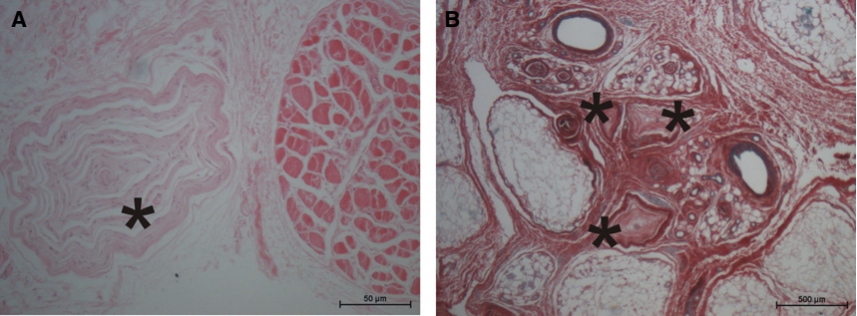
(A) Pacinian body (asterisk) next to a muscle fiber bundle of the palmaris brevis muscle (HE staining; 78‐year‐old male donor). (B) Pacinian bodies (asterisks) without association to muscle fibers (Sirius red staining; 94‐year‐old female).

Single Pacinian bodies were frequently seen on palmaris brevis muscle sections. In most of the cases, they were some distance from the muscle fiber bundles and did not show a direct morphological connection to either the muscle fibers or their tendon‐like extensions. In a few cases, the Pacinian body was close to a muscle fiber bundle but, again, without specific fibrous connection (Fig. 4).

The topographic location of the Pacinian bodies with respect to the palmaris brevis muscle, were mainly at the dorsal side of the muscle towards the palmar aponeurosis. However, palmar‐located Pacinian bodies might have been removed due to the preparation of the muscle. Therefore, a complete statement on the distribution of Pacinian bodies next to the palmaris brevis muscle was not possible from our samples.

## Discussion

The human palmaris brevis muscle morphology showed some general and some individual aspects that should be considered when talking about this muscle.

In general, the palmaris brevis shows no connection to the dermis; in accordance with the new literature (Kim et al. 2017) the muscle fibers originated from the palmar aponeurosis and inserted in the fascia of the hypothenar muscle. The size of the muscle in our sample was comparable to the literature (Kim et al. 2017); the small differences in the muscle length were due to a measurement of the actual fiber length we used in our setting. In a single individual (an 88‐year‐old male), we noted an increase in muscle width. This phenomenon was also seen by others (Nayak & Krishnamurthy, 2007; Kim et al. 2017); we would rather term it an individual variation than a muscle hypertrophy as suggested by others (Nayak & Krishnamurthy, 2007). In addition, we observed another individual variation, namely a smaller developed palmaris brevis which was present in a number of female donors. Following the idea of Moore & Rice (2017) that the palmaris brevis has a proximal and a distal portion, the short variant we observed could be characterized as a lack of the distal part in these individuals. Interestingly, the case in our sample with partial muscle atrophy (77‐year‐old male) showed that the atrophic fibers were only located in the distal portion, whereas the proximal portion was well preserved. Since we did not find atrophic muscle tissue in the female cases with smaller developed palmaris brevis muscle, we suspected rather an inherent variation than a degenerative process.

Concerning the neuronal sensors of the palmaris brevis muscle it is interesting to note that muscle spindles were never counted in this muscle (Voss, 1971). Our findings revealed a high number of muscle spindles that exceeded the expected number calculated with the formula of Banks (2006) by a factor of 1.7. Thus, the human palmaris brevis muscle shows all structural components necessary for reflex control of its length and might therefore include not only voluntary control (Moore & Rice, 2017) but also some reflex stability. This might also explain the clinical observed palmaris brevis spasm (Serratrice et al. 1995; Liguori et al. 2003; Tarsy et al. 2004; Eswaradass et al. 2014; Arányi & Kovács, 2015; Yaacob & Yeo, 2017). Interestingly, we could not detect any sensors detecting changes in muscle strength (Golgi tendon organs, Ruffini‐like corpuscles, free nerve endings in the musculo‐tendinous junction). This might indicate that this parameter is not necessary in the palmaris brevis, a muscle with no connection to skeletal structures. A second example for this type of muscle is the platysma, where also no Golgi tendon organs or Ruffini‐like corpuscles were observed (May et al. 2018). Another suggestive explanation might be connected to the high number of compound spindles (in comparison with vastus or peroneus longus – Watanabe & Suzuki, 1999). Although there are no ideas in the literature as to whether the morphology of compound spindles might influence responsiveness, the v‐shaped arrangement of muscle spindles might induce some pressure/tension‐induced sensory signals replacing the specific corpuscular sensors usually present.

Many Pacinian corpuscles are described in the human palm (Stark et al. 1998; Kobayashi et al. 2018; Rhodes et al. 2019) but there was no specific association between Pacinian corpuscles and the palmaris brevis muscle in our samples.

## Conflicts of interest

No author has any conflicts of interest.

## Author contributions

C.A.M. took tissue samples, performed the evaluation, and prepared the article. S.B. performed the staining and reviewed the article.
